# Activation of cAMP (EPAC2) signaling pathway promotes hepatocyte attachment

**DOI:** 10.1038/s41598-023-39712-3

**Published:** 2023-07-31

**Authors:** Grace Aprilia Helena, Teruhiko Watanabe, Yusuke Kato, Nobuaki Shiraki, Shoen Kume

**Affiliations:** 1 School of Life Science and Technology, Tokyo Institute of Technology, 4259-B-25 Nagatsuta-cho, Midori-ku, Yokohama, Kanagawa 226-8501 Japan; 2 Life Science Laboratory, Technology and Development Division, Kanto Chemical Co., Inc., 21 Suzukawa, Isehara, Kanagawa 259-1146 Japan

**Keywords:** Cell biology, Cell adhesion, Cell growth, Cell signalling

## Abstract

Primary Human Hepatocyte (PHH) remains undefeated as the gold standard in hepatic studies. Despite its valuable properties, partial attachment loss due to the extraction process and cryopreservation remained the main hurdle in its application. We hypothesized that we could overcome the loss of PHH cell attachment through thawing protocol adjustment and medium composition. We reported a novel use of a medium designed for iPSC-derived hepatocytes, increasing PHH attachment on the collagen matrix. Delving further into the medium composition, we discovered that removing BSA and exposure to cAMP activators such as IBMX and Forskolin benefit PHH attachment. We found that activating EPAC2, the cAMP downstream effector, by S-220 significantly increased PHH attachment. We also found that EPAC2 activation induced bile canaliculi formation in iPS-derived hepatocytes. Combining these factors in studies involving PHH or iPS-hepatocyte culture provides promising means to improve cell attachment and maintenance of hepatic function.

## Introduction

The liver holds a prodigious role in maintaining body homeostasis. It performs myriads of immensely complex metabolic functions crucial for life. It also orchestrates a wide array of metabolic processes in the human body, such as xenobiotic clearance, fat metabolism, and blood albumin production^1^. Hepatic studies in the past mainly relied on animal and hepatoma cell lines as the model subjects^2^. However, these subjects cannot completely simulate the human liver response to drug insults, hampering the progress in hepatic studies^3^.

Primary Human Hepatocyte (PHH) is the gold standard for a model subject in hepatic studies due to its ability to demonstrate similar *in-vivo* responses to drug insults^3,4^. Previous works have used PHH as the primary research subject, highlighting it as an indispensable model for pharmacological studies^1^. Despite its significance, PHH availability remains the main problem hindering hepatic studies. PHH is bounded to donor availability which causes variances between batch-to-batch products^5^. Furthermore, the harsh isolation process from the donor’s liver inflicts stress upon the cells, which leads to a partial loss of cell attachment and function^4^. Isolated hepatocytes go through the cryopreservation process, which further reduces their viability and cell attachment despite the methods developed over the years^6^.

Cell–matrix attachment is an essential element in maintaining hepatic function and differentiation state. It is not only crucial in the 2D culture format but also required for hepatocyte spheroid formation^7^. Loss of cell–matrix attachment affected the hepatocyte differentiation state and its ability to undergo long-term culture *in vitro*^6^. Therefore, reverting cell attachment of cryopreserved PHH is crucial to ensure its versatility in hepatic studies.

cAMP (cyclic AMP) is a second messenger that mediates a wide range of cellular processes^8^. Two main effectors further relay the cAMP signal: PKA (Protein Kinase A) and EPAC (Exchange Proteins directly activated by cAMP)^9^. A considerable amount of literature has reported the role of cAMP in mediating cell–matrix attachment^10,11^. However, these studies do not use hepatocytes as the subject and primarily focus on elucidating cAMP-dependent PKA (Protein Kinase A). Recent attention has been paid to EPAC to elucidate the mechanism of cell–matrix attachment^12,13^. EPAC possesses two main isoforms, EPAC1 and EPAC2^9^. The expression of these isoforms differs according to the cell type. The sub-isoform of EPAC2, EPAC2C, is exclusively expressed in hepatocytes^14^. Despite the peculiarity of EPAC2 expression in hepatocytes, studies in this field are mainly focused on the link between EPAC1 and cell attachment^12,15^. Hence, not many studies explore the role of EPAC2 in hepatic function and attachment.

Here, we report an attempt to improve cell attachment and hepatic function using a medium designed for iPSC-derived hepatocytes and protocol optimization. We used suspension PHH as a model to simulate post-thaw cell attachment. We further discovered that cAMP activation by IBMX and Forskolin improved cell attachment in PHH. We demonstrated that EPAC2 activation potently improved cell attachment in PHH and induced bile canaliculi formation in iPS-derived hepatocytes.

## Results

### Successful hepatic differentiation of human iPSCs with a chemically defined maturation medium

**Figure 1:**
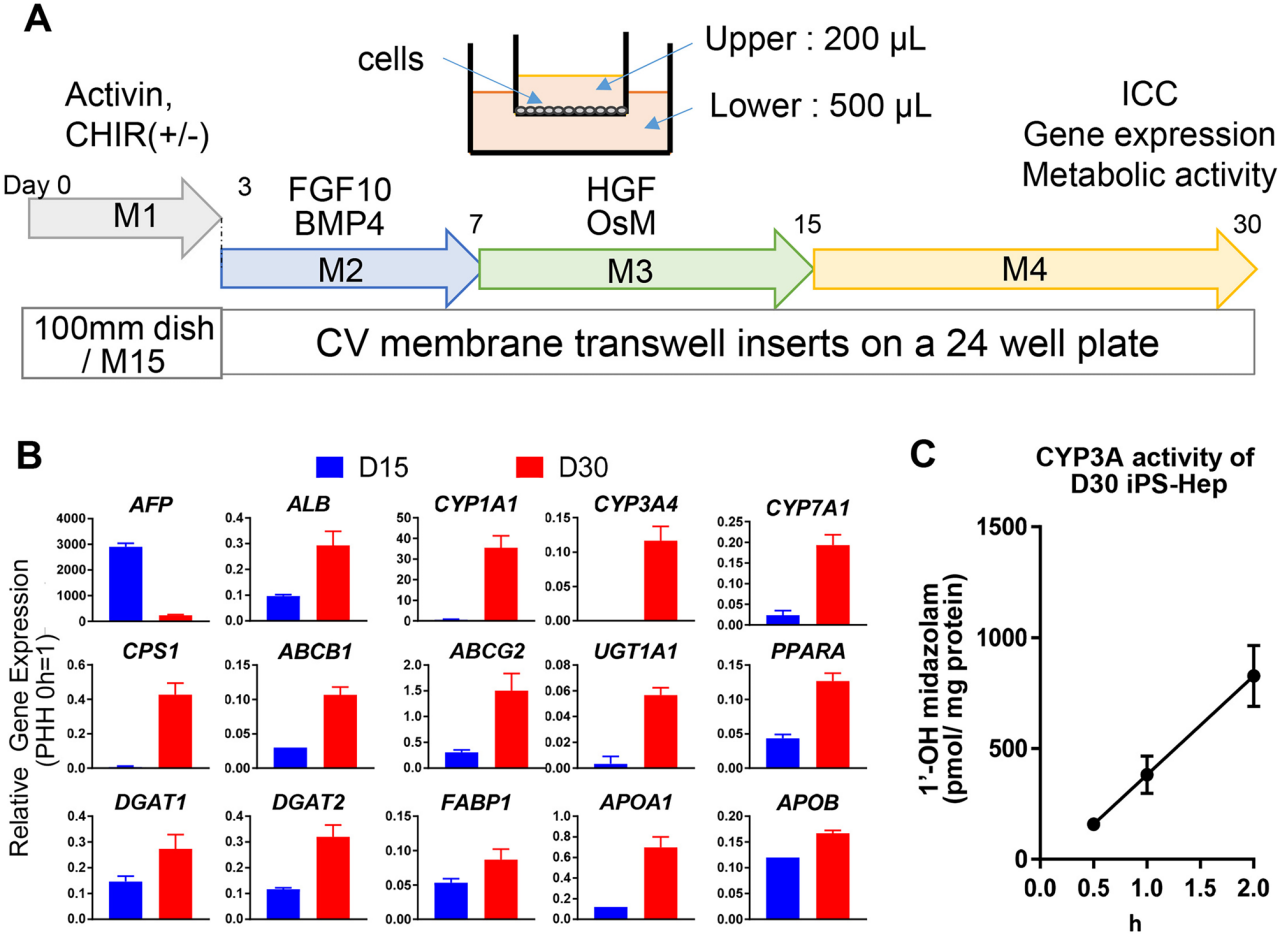
Successful hepatic differentiation of human iPSCs with a novel maturation medium. (**A**) Schematic illustration of hepatic differentiation of hiPS cells. We used a novel M4 medium to obtain hiPS-hep. (**B**) iPS cell-derived hepatic precursor cells on differentiation day (**D**) 15 (blue bars), and iPS-hep on D30 (red bars) were analyzed for mature hepatocyte marker expressions by q-PCR analysis (n = 3). Note that the expression levels are relative versus primary hepatocytes (PHH) at 0 h (= 1). (**C**) D30 iPS-hep exhibited CYP3A activity (n = 3).

We previously reported a culture system for human induced pluripotent stem cells (hiPSCs) into hepatocyte-like cells using a commercial maturation medium (Nakai et al.^40^). We then sought to establish a chemically defined maturation medium (M4 medium) for iPS-hep differentiation (Fig. 1). We performed hepatic differentiation using cryo-stocked definitive endoderm (hiPS-endo) cells derived from RPChiPS771. Thereafter, the differentiation was performed on a Collagen Vitrigel (CV) membrane in a transwell culture format (Fig. 1A). hiPS-endo were cultured with M2 medium from days 3–7, then changed into M3 medium on days 7–15. From day 15 onwards, the medium was switched to an M4 maturation medium.

Day 30 iPS-hep (iPS-derived Hepatocyte) RT-PCR result revealed a reduced expression of AFP (Alpha-fetoprotein) and increased expression of mature hepatocyte markers compared to day 15. *ALB* (Albumin), phase I drug-metabolizing enzymes (*CYP1A1*, *CYP3A4*, and *CYP7A1*), conjugating enzyme (*UGT1A1*), drug transporter enzymes (*ABCB1* and *ABCG2*), lipid metabolism genes (*DGAT2*, *FABP1*, *APOA1*, and *APOB*) showed increased expression compared to those of day 15 iPS-derived hepatic precursor cells (Fig. 1B). The expression levels are shown as relative levels versus primary hepatocytes (PHH) at 0 h (= 1). *ALB* expression was approximately 0.3-fold of the PHH. *CYP3A4* was ~ 0.12-fold of PHH. D30 iPS-hep can convert midazolam to its metabolite (1’-OH midazolam), demonstrating their CYP3A activity (Fig. 1C). Therefore, our results indicate that the maturation medium M4 is useful for the maturation into iPS-hep.

### PHH cultured in the M4 medium showed better cell attachment than in other commercial media

**Figure 2:**
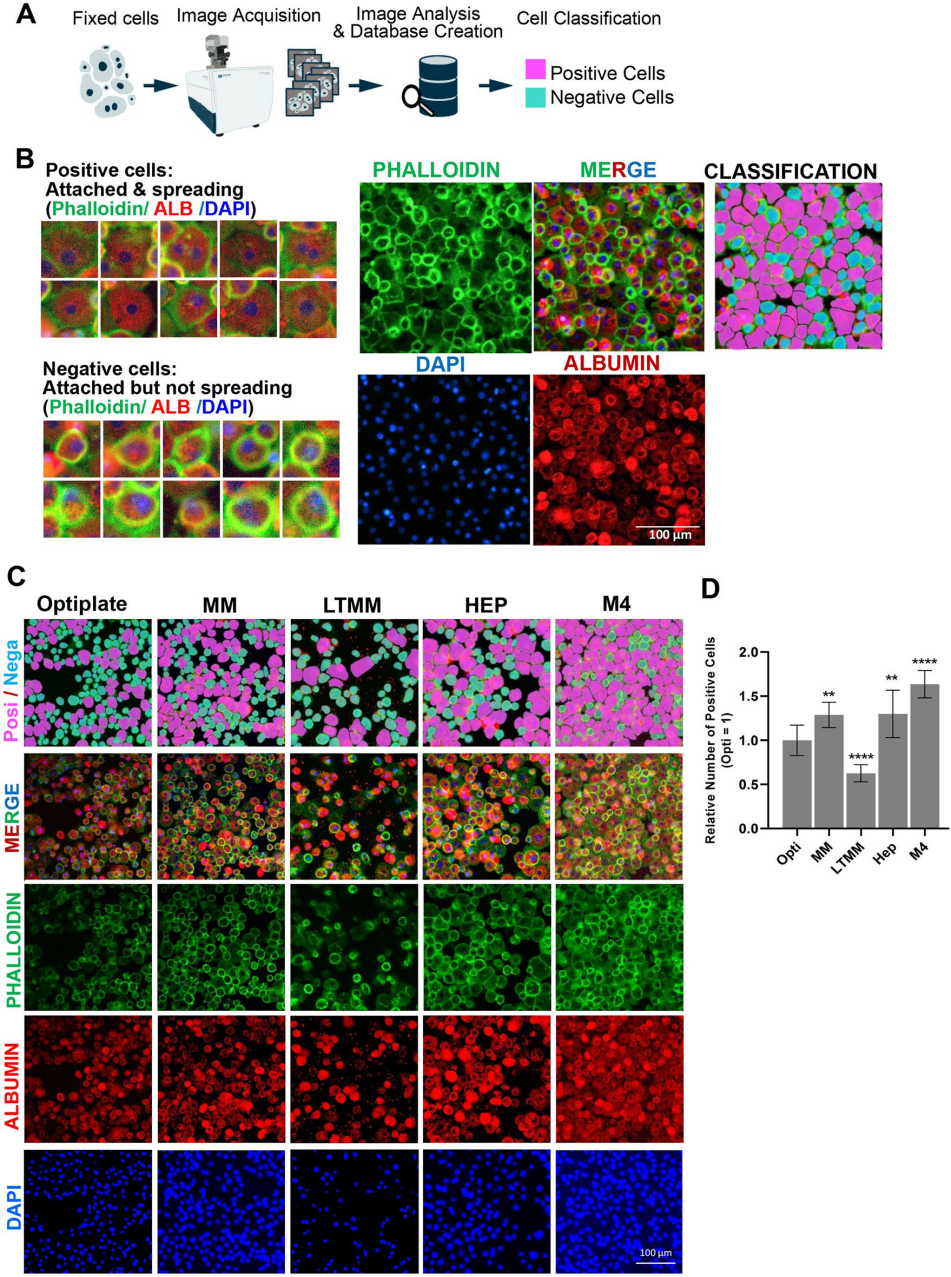
PHH in M4 showed better cell adhesion than other commercial media. (**A**) Schematic illustration of staining, image quantification, and classification. (**B**) (Left) Manual image training characterization for machine learning and automated quantification. The identified cells are classified into two groups: “positive” cells that appear to be attached & spreading (upper left) and “negative” cells that appear to be attached but non-spreading (lower left). (Middle) Representative staining images with Phalloidin (green), anti-ALB (red), DAPI (blue), and merged images are shown. (Right) The classification diagram illustrating the “positive” (magenta) and “negative” (light blue) cells are shown in the middle images. Note that the positive cells (attached & spreading) are larger and show faint peripheral phalloidin staining. The negative cells (attached but non-spreading) are smaller, round-shaped, and show strong peripheral phalloidin staining. (**C**) PHH cultured for 4 h (h) in M4 medium or several other commercial media, Optiplate, MM, LTMM, and HEP are shown. PHH was stained with Phalloidin (green). The classification diagrams illustrating the “positive” (magenta) and “negative” (light blue) cells are shown in lower panels. (**D**) Quantification of the staining shown in (**A**) and (**B**). Data are expressed as mean ± SD, n = 10. N is the total number of views (images), with 3–5 views (images) /well (treatment) × 3–5 wells (treatments). The relative number of positive cells was obtained by dividing the average positive cell number (per image) by that of the control treatment. Differences are shown as **p < 0.01 or ****p < 0.0001; analyzed with one-way ANOVA, Dunnett’s multiple comparisons tests.

In our preliminary experiments, PHH cultured in the M4 medium demonstrated improved cell attachment to other media (GH unpublished). We established an image-based cell attachment quantification method from the immunostaining images (Fig. 2A, B). The images were acquired with MetaXpress and analyzed with CellProfiler to create image data containing all the measurements. The image data were further analyzed with CellProfiler Analyst in the cell classifier module to manually classify the cells into positive (attached and spreading cells) and negative (attached but non-spreading cells) as samples for further quantification by machine learning.

We manually identified positive cells by observing the morphology of PHH with phalloidin staining. Phalloidin stains the cytoskeleton (F-actin) on the peripheral area of the hepatocytes^16,17^. Attached and spreading PHH displayed pebble stone-like morphology, closely connecting to the neighboring cells. We call these “positive cells” (Supplementary Fig. S1; Fig. 2B, magenta)^16–19^. Conversely, attached but non-spreading cells demonstrated circular morphology with high intensity of phalloidin staining; we identified them as “negative cells” (Supplementary Fig. S1; Fig. 2B, green). However, as Phalloidin only stains the peripheral area of the cells, we used ALB to identify hepatocytes, optimizing cell identification with machine learning. As ALB localized to the cytoplasm, anti-ALB stained the cytoplasm of PHH and improved software-based identification. DAPI staining was helpful for the comprehensive quantification of the cell. The merged staining images identified the “positive cells” (attached and spreading cells) and “negative cells” (attached but non-spreading cells). The software classifies the identified cells within the database based on the manual training input and automatically classifies the rest of the identified cells into “positive” and “negative” cells.

Experiments with our M4 medium showed promising results on iPS-hep differentiation. We then used this M4 medium in culturing PHH and compared the cell attachment with five types of commercially available media: M4, MM, LTMM, Hep, and Optiplate. Compared to other commercial media, the hepatocytes cultured in our M4 medium showed improved cell attachment, sufficient spreading, proper cell-to-cell connections, and even monolayer formation (Fig. 2C). Quantification results revealed that PHH cultured in our M4 medium showed significantly higher “positive” (attached and spreading) cells than the other commercial media (Fig. 2D). These results suggest that our M4 is superior in improving early hepatocyte attachment.

### Low BSA concentration improved PHH attachment

**Figure 3:**
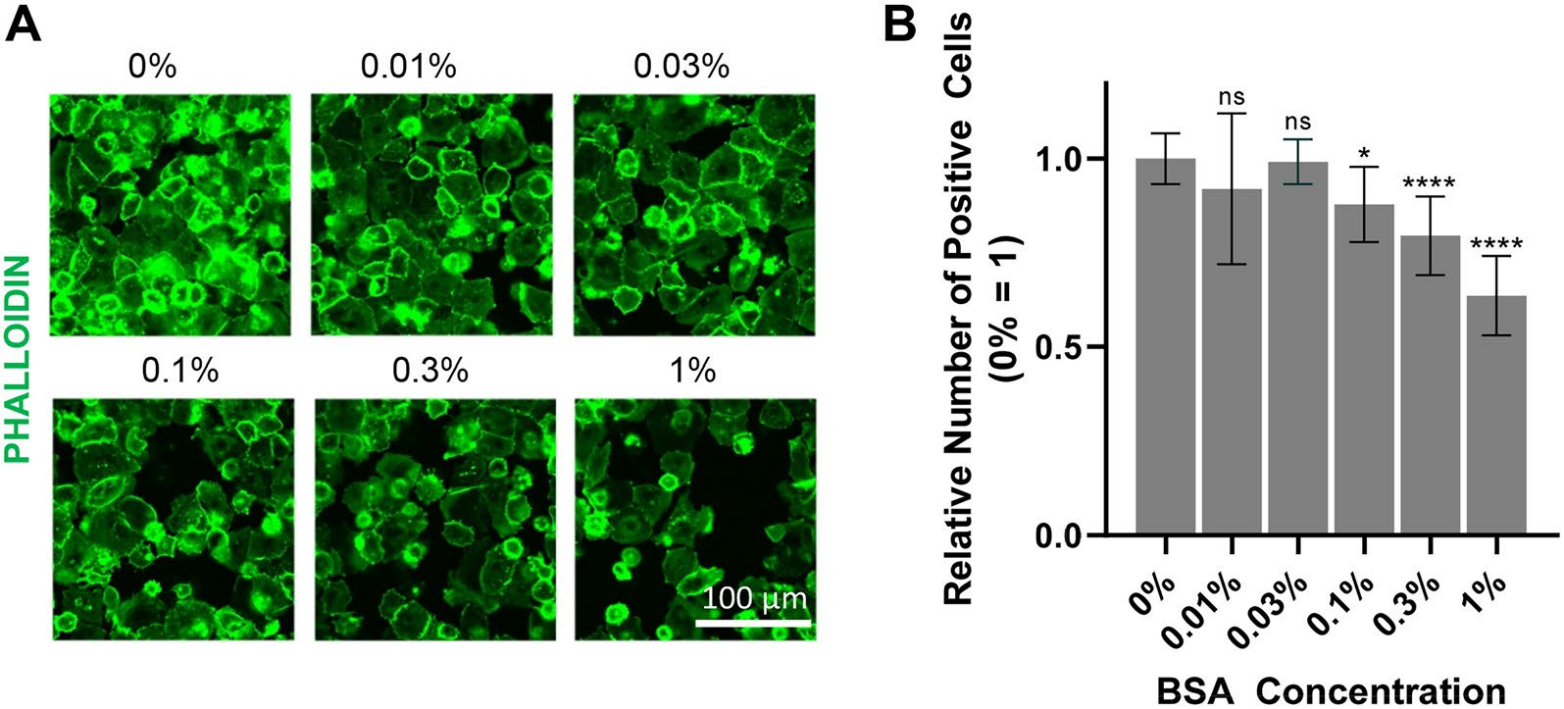
Low BSA concentration improved PHH attachment. (**A**) Phalloidin staining (green) of 4 h PHH H789 cultured in the M4 medium with different BSA concentrations. (**B**) Quantification result of phalloidin staining. Data are expressed as mean ± SD, n = 10. Differences are shown as *p < 0.05 or ****p < 0.0001; analyzed with one-way ANOVA, Dunnett’s multiple comparisons tests.

We investigated the effect of the bovine serum albumin (BSA) concentration on PHH cell attachment in our M4 medium to enhance the adhesion efficiency and reduce xenobiotic components in the medium composition. We tested the BSA concentration that affected PHH cell attachment in our M4 medium. We plated the cells in M4 BSA-free (BSA–) medium with various concentrations of BSA from 0 to 1%. We observed the highest average number of relative positive cells for cells cultured in 0% BSA (BSA–) condition. On the contrary, cells cultured in M4 medium with 1% BSA concentration showed the lowest relative positive cell number in comparison to lower BSA concentrations (Fig. 3A, B). In short, we observed a decrease in the number of attached and spreading cell numbers as the BSA concentration increased. Therefore, this result confirms that lower BSA concentration improved PHH attachment.

### Seeding density is an essential factor in PHH attachment

**Figure 4:**
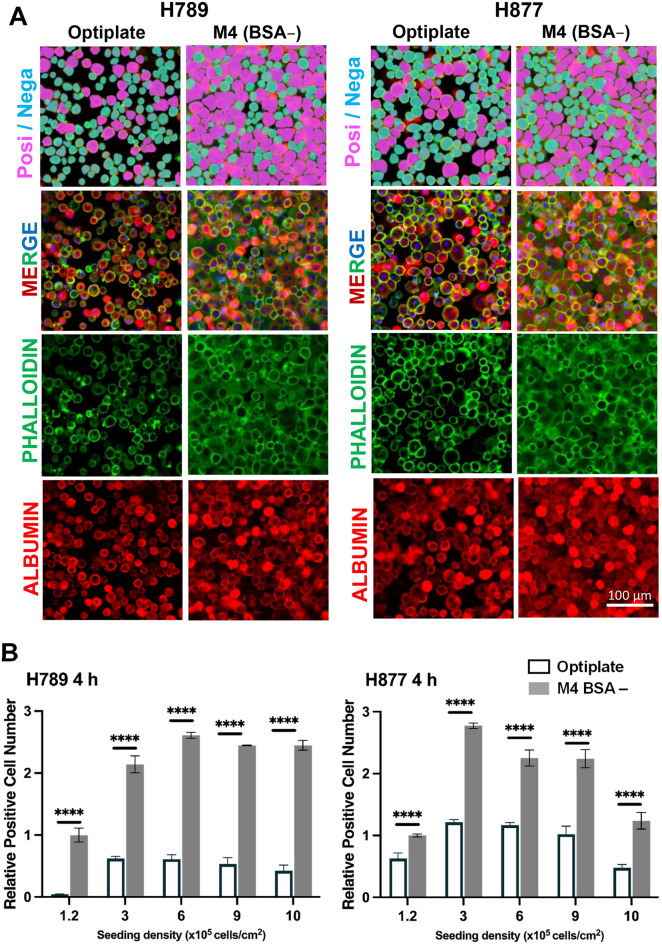
Seeding density is an essential factor in PHH attachment. (**A**) Phalloidin staining (green) of PHH H789 and H877 in M4 and Optiplate, at 4 h post-plating at a seeding density of 6 × 10^5^ cells/cm^2^. (**B**) Quantification result of phalloidin staining. Data are expressed as mean ± SD, n =3. Differences are shown as ***p < 0.001 or ****p < 0.0001; analyzed with two-way ANOVA, Sidak’s multiple comparisons tests.

We performed PHH culture with two lots of suspension type PHH (H789 and H877) in different seeding densities ranging from 1.2 × 10^5^ to 1 × 10^6^ cells/cm^2^ in Optiplate (negative control), and our M4 BSA– medium. The cells were incubated for 4 h. Phalloidin staining revealed that the cells cultured in our M4 BSA– medium had a better attachment and spreading morphology than those in the Optiplate medium (Fig. 4A). From the phalloidin staining result, it was observed that different lots of PHH possessed different attachment capabilities and optimal seeding densities. H877 demonstrated better cell adherence and spreading compared to H789 in the Optiplate medium. H789 plated at 6 × 10^5^ cells/cm^2^ showed the highest relative number of positive cells compared to the other seeding density except for H877 (Fig. 4B). H877 exhibited the highest relative positive cell number at 3 × 10^5^ cells/cm^2^. Cells in higher densities (9 × 10^5^ cells/ cm^2^ and 1 × 10^6^ cells/ cm^2^) demonstrated reduced attached cell numbers. The results confirmed that removing BSA and optimizing seeding density substantially enhance PHH cell attachment.

### cAMP activators improved PHH attachment and function in Long-Term culture

**Figure 5:**
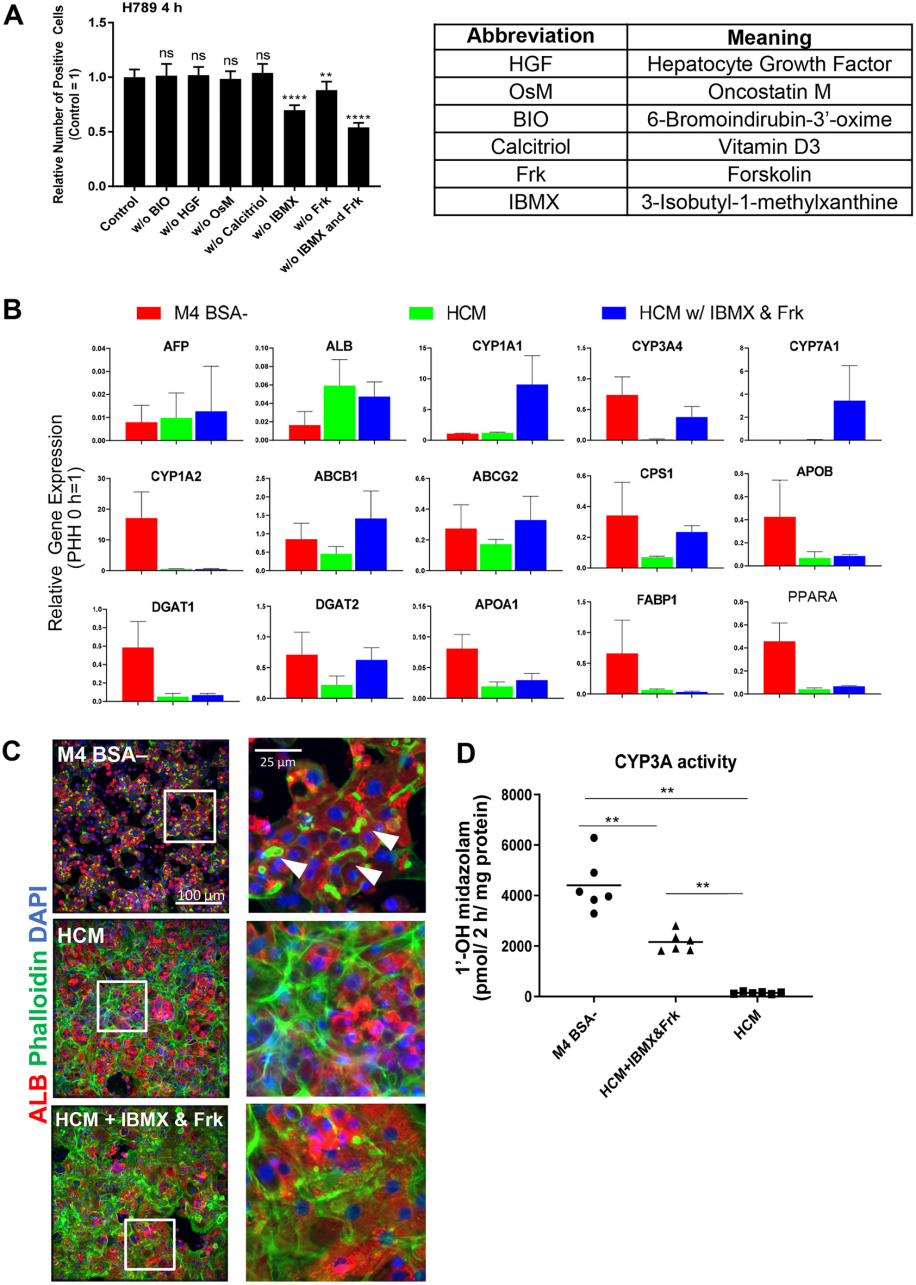
cAMP activators improved PHH attachment and function in long-term culture. (**A**) Quantification result of PHH in M4 BSA– with component eliminations. Differences are shown as **p < 0.01 or ****p < 0.0001; analyzed with one-way ANOVA, Dunnett’s multiple comparisons tests. (**B**) qPCR result of PHH day 7 cultured in M4 BSA– (red bars), HCM (green bars), and HCM w/ IBMX and Frk (blue bars). Data are expressed as mean ± SD, n = 3. White arrow heads depict bile canaliculi structures. (**C**) Staining images of PHH day 7 cultured in M4 BSA–, HCM, and HCM w/ IBMX and Frk with anti-ALB antibody (red), Phalloidin (green), and DAPI (blue). White arrows marked the bile canaliculi structure in between the hepatocytes. (**D**) CYP3A activity of PHH measured by midazolam metabolite on day 7 after plating in M4 BSA–, HCM, and HCM w/ IBMX and Frk. Data are expressed as mean ± SD, n = 3. Differences are shown as **p < 0.01, analyzed with one-way ANOVA Dunnett’s multiple comparisons tests.

We then investigated the component in our M4 medium that positively affects PHH attachment. The list of M4 components can be viewed in (Fig. 5A, right). PHH were cultured in M4 BSA– medium. To identify the components responsible for improving PHH attachment, we removed a single or a combination of components from the M4 medium. The relative number of positive cells (attached and spreading cells) decreased to approximately 70% and 80% of the control (M4 BSA–) when cultured without either 3-isobutyl-1-methylxanthine (IBMX) or Forskolin and decreased to approximately 50% when both IBMX and Forskolin were removed (Fig. 5A, right). Because IBMX and Forskolin target the cAMP pathway, we further investigated the effect of cAMP effector activation and inhibition^9^.

After confirming the positive impact of cAMP activators (IBMX and Forskolin) on PHH attachment and adhesion, we further assessed their effects in long-term PHH culture (7 days). We compared PHH cultured in our present M4 BSA– medium with another commercially available HCM medium. We added IBMX and Forskolin to HCM as an additional treatment. RT-PCR results revealed that PHH in BSA– (Fig. 5B, red bars) showed less expression of AFP and ALB compared to other treatments. However, cells in this treatment showed higher relative gene expression of P450 enzymes such as *CYP3A4* and *CYP1A2* compared to the other treatments. Furthermore, cells in this treatment also demonstrated better expression of genes involved in fat metabolisms, such as *PPARA*, *FABP1*, *DGAT1*, *DGAT2*, and *APOA1*. Expressions of genes required in urea synthesis (*CPS1*) and cholesterol transport (*APOB*) were also the highest in this condition*.* In short, cells in this condition demonstrated metabolic gene expression patterns close to 0 h Suspension PHH (0 h Suspension PHH = 1). In HCM with IBMX and Forskolin treatment (Fig. 5B, blue bars), transporter genes (*ABCB1* and *ABCG2*) and other CYP genes (*CYP1A1* and *CYP7A1*) were highly expressed. The results imply that adding IBMX and Forskolin to HCM enhances cell metabolism.

PHH cultured in M4 BSA– condition showed distinguishable individual cell shapes compared to other treatments from the Phalloidin staining at the cell periphery (Fig. 5C). We observed the possible structure of hepatic bile canaliculi formation indicated by the high intensity of actin staining between the cell–cell connections (white arrows). We further confirmed that the bile canaliculi structure by phalloidin staining was also colocalized with MRP2 (Supplementary Fig. S2A). These results further confirmed the presence of bile canaliculi structure in long-term culture.

Cells cultured in HCM medium with IBMX and Forskolin showed different cell morphology than HCM (Fig. 5C). Cells in HCM exhibited an abundance of actin stress fibers revealed by phalloidin staining. In this condition, we observed the aberration of cellular structure deduced from the undistinguishable individual cell structure. In HCM with IBMX and Forskolin condition, although expressing such an abundant amount of actin stress fiber, the individual cells showed more distinguishable morphology than HCM treatment alone.

CYP3A activity assay result revealed that PHH in M4 BSA– condition showed the highest CYP3A activity (Fig. 5D). In supporting the result, adding IBMX and Forskolin into the HCM enhances CYP3A4 activity compared to the HCM medium alone. At the same time, adding IBMX and Forskolin to HCM showed overall better early PHH attachment, prevented over-spreading and stress fiber formation in long-term culture, and improved hepatic gene profile and metabolism.

### EPAC2 activation potently induced PHH attachment

We assayed their viability as PHH tends to dedifferentiate in the long-term culture. Understanding the importance of BSA in culture medium, we hypothesized that BSA addition might be detrimental to PHH in the long-term culture. Therefore, we compared BSA+ and BSA– treatments. Live/dead assay revealed that approximately 70% of the PHH cultured in both conditions were viable (Supplementary Fig. S2B). The low PH3 ratio ranging from 1 to 3% suggests that minimum proliferation rate of PHH (Supplementary Fig. S2C). Variance observed in Albumin secretion of PHH cultured under the M4 BSA– condition compared to those in M4 BSA+ suggests that culturing PHH under M4 BSA– condition might not be suitable for long-term culture (Supplementary Fig. S2D).

**Figure 6:**
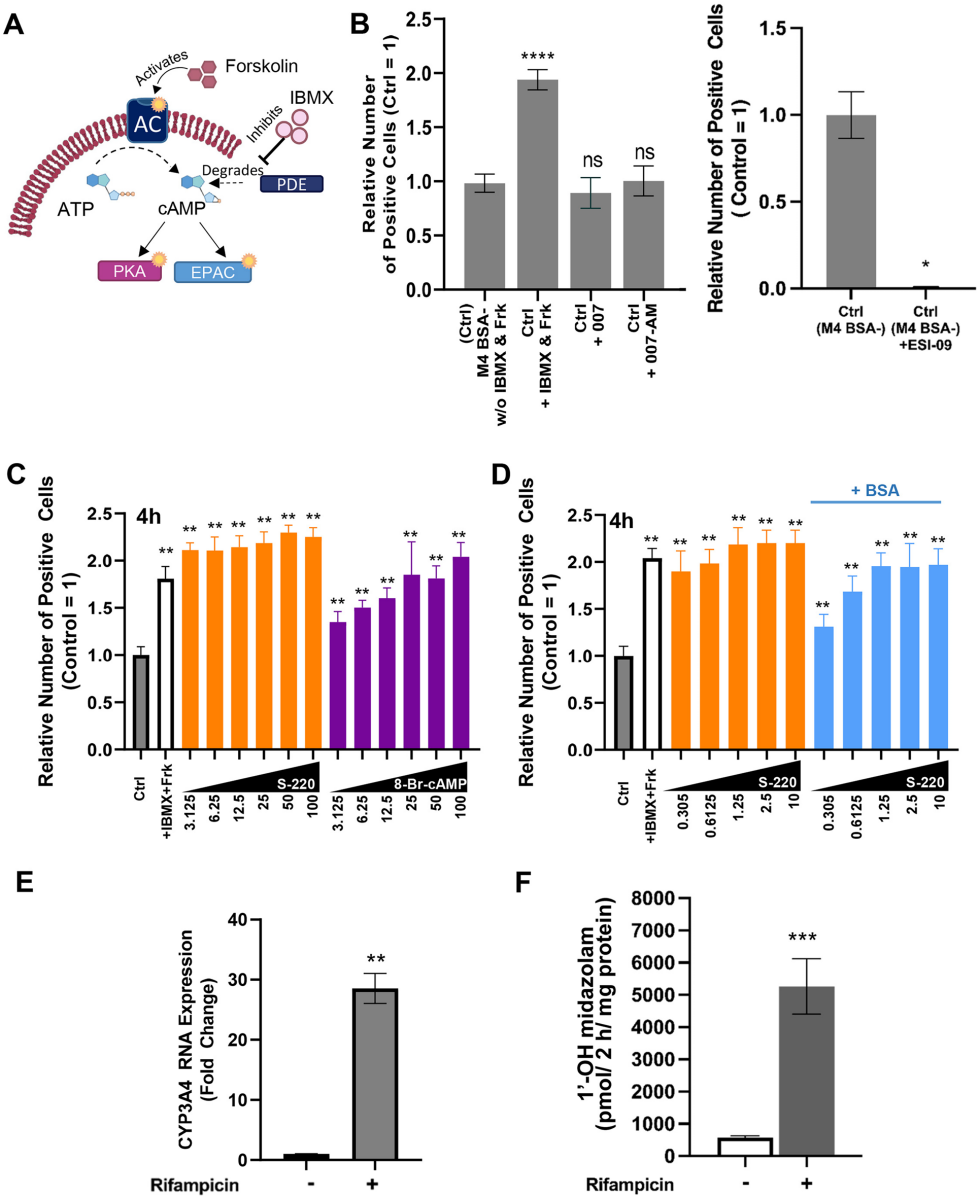
EPAC2 Activation Potently Ignited PHH Attachment. (**A**) Illustration of cAMP activation by IBMX and Forskolin in cells. (**B**) Quantification results of phalloidin staining at 4 h post-thaw PHH in M4 BSA– (Ctrl: control), Ctrl + IBMX and Frk, Ctrl + 007, and Ctrl + 007-AM (left) or Ctrl + ESI-09 (6.25 µM) (right) (n = 15). (**C**, **D**) Quantification results of phalloidin staining of PHH at 4 h post-thaw in ctrl (grey bars, negative control), + IBMX and Frk (white bars, positive control), M4 BSA– added with S-220 (orange bars) or 8-Br-cAMP (purple bars), or M4 BSA + added with S-220 (blue bars) (D). Data are expressed as mean ± SD, n = 10 (**B**, **C**), and n = 14 (**D**). (**E**, **F**) The induction of *CYP3A4* expression (**E**) and CYP3A activity (**F**) by rifampicin, a known CYP3A inducer. (**B** left, **C**, **D**) Differences are shown as **P < 0.01 or ****P < 0.0001 analyzed with one-way ANOVA Dunnett’s multiple comparisons tests. (B right, E, F) *p < 0.05, **P < 0.01, ***P < 0.001; analyzed with Student’s t-test.

The cAMP signal is further relayed by its two main effectors, EPAC and PKA (Fig. 6A). A number of studies have investigated the role of EPAC activation in improving cell adhesion by adding the EPAC1 activator, 007^13^. We then exposed PHH to EPAC1-specific activators (007 and 007-AM). 007 addition to M4 BSA– without the presence of IBMX and Forskolin did not improve PHH attachment (Fig. 6B, left). We then treated PHH with an EPAC2-specific inhibitor (ESI-09) in M4 BSA– without IBMX and Forskolin, which caused a reduction in the relative number of cell attachments (Fig. 6B, right). ESI-09 inhibits both isoforms of EPAC (EPAC1 and EPAC2). The result led us to deduce that EPAC2, but not EPAC1, might control PHH attachability.

We then exposed PHH to M4 BSA– medium with the activator of EPAC2 (S-220) and PKA (8-Br-cAMP) in a serially diluted concentration from 3.125 µM to 100 µM (Fig. 6C). Although 8-Br-cAMP (Purple bars) was effective in high concentrations, cells exposed to S-220 (Orange bars) showed higher cell attachment even at the lowest concentration (3.125 µM) (Fig. 6C). These results indicate a high degree of EPAC2 involvement in regulating PHH attachment.

The lowest concentration of S-220 was proven to increase cell attachment. Further experimentation was performed by subjecting PHH to an even lower concentration of S-220 (0.3–10 µM) in both BSA– and BSA+ conditions (Fig. 6D). Quantification of 4 h post-thaw images revealed that even the lowest concentration of S-220 (0.3 µM) (orange bars) maintained the number of positive cells in BSA– treatment close to the positive control (white bar, w IBMX & Frk). In contrast, the cells in the BSA+ medium showed a lower positive cell number than BSA– (blue bars) but a relatively higher number than the negative control (grey bar). This result confirmed that EPAC2 potently improves PHH attachment in BSA– conditions.

We then confirmed the induction of CYP3A expression and CYP3A activity by rifampicin, a known CYP3A inducer. We cultured the PHH in M4 BSA– condition for 2 days, then changed to medium (BSA+) for the induction and metabolism assays. We confirmed that rifampicin addition induced both the expression of *CYP3A4* (Fig. 6E) and metabolizing enzyme activity, assayed by 1’-OH midazolam generation (Fig. 6F). Therefore the expression and activity of CYP3A4 were inducible in the attached PHH.

### EPAC2 activation in iPS-hep improved bile canaliculi formation

**Figure 7:**
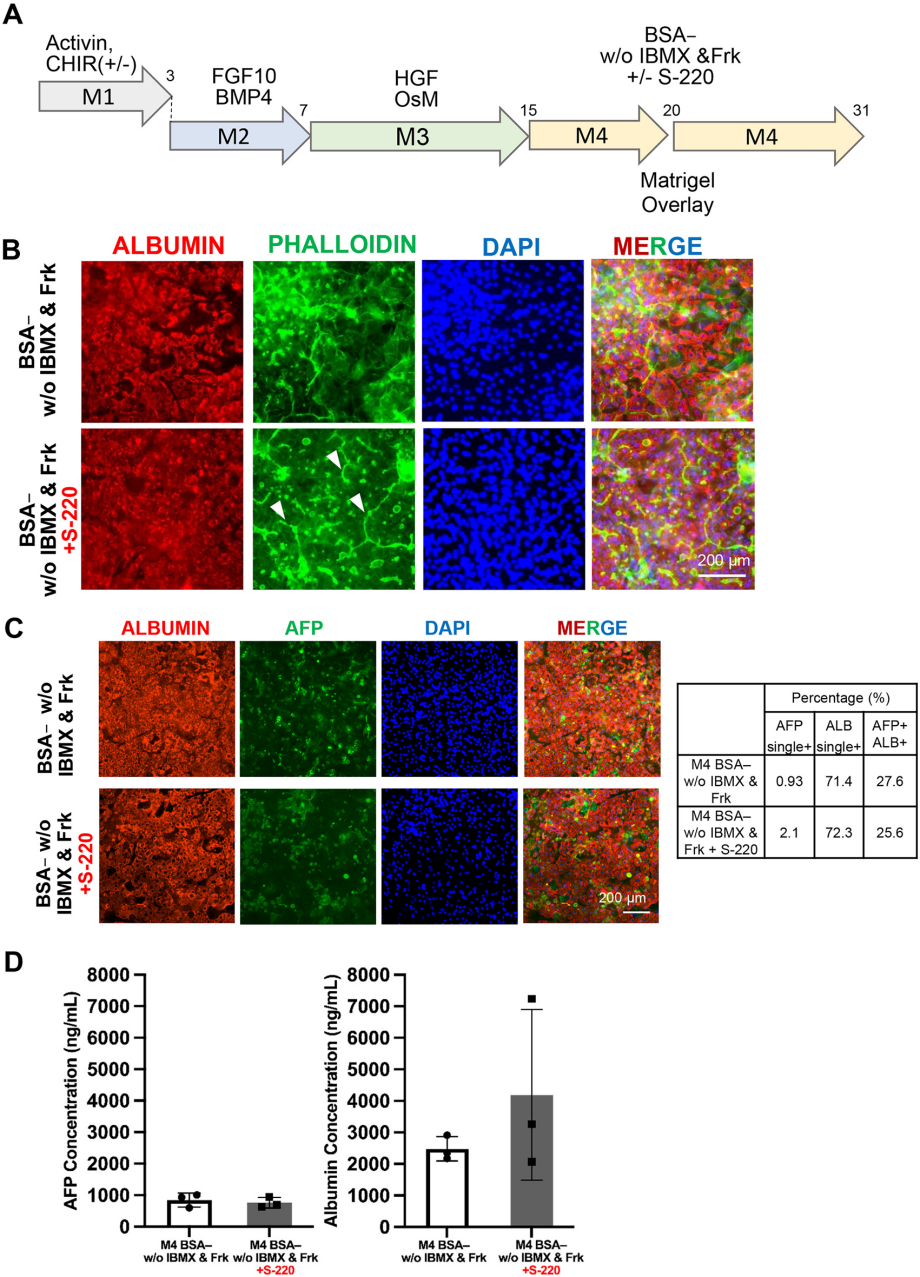
EPAC2 Activation in iPS-Hep improved Bile Canaliculi Formation. (**A**) Differentiation scheme of hepatic differentiation from iPSC. (**B**) Immunostaining of D31 iPS-hepatocytes with Albumin (red), Phalloidin (green), and DAPI (Blue). (**C**) (left) Immunostaning images of D31 iPS-Hep with Albumin (red), Alpha-fetoprotein/AFP (green), and DAPI (blue) and MERGE. (Right) A summary table of Immunocytochemistry quantification for average ALB (Albumin), AFP (Alpha-fetoprotein) single positive and double positive (n = 8). (**D**) AFP and ALB secretion of D31 iPS-Hepatocyte (n = 3).

We hypothesized that besides regulating hepatocyte attachment, EPAC2 might be more prominent in orchestrating overall hepatic function. Past studies have revealed that EPAC isoforms showed organ-specific expression. The EPAC2 isoform, EPAC2C, is solely expressed in the liver. To elucidate the role of EPAC2 in differentiation, we performed hepatic differentiation from iPS and added an EPAC2 activator (S-220) in our M4 BSA– medium from day 15 until day 30 of the differentiation. Since it is well known that the sandwich configuration enhanced the morphology and viability of hepatocytes and helped the formation of bile canaliculi structures^20,21^, we performed matrigel overlay onto the iPS-Hep on day 20 (Fig. 7A).

Immunostaining result of Phalloidin revealed the formation of bile canaliculi structure in S-220 treated day 31 iPS-hep, indicated by a high intensity of actin staining between the cell–cell connections (Fig. 7B, white arrows). CDFDA staining further confirmed the formation of bile canaliculi under S220 addition compared to the control M4 BSA– condition (Supplementary Fig. S3). The result indicated an unexpected role of EPAC2 activation in the bile canalicular formation of iPS-derived hepatocytes. Using our M4 BSA– condition, we could generate approximately 71% iPS-Hep that were ALB-single positive (Fig. 7C). The iPS-Hep showed a lower AFP secretion with high ALB secretion on day 31 of differentiation (Fig. 7D). Therefore, these results suggest that S-220 addition did not seem to negatively affect parameters such as AFP/ALB secretion, and improved bile canaliculi formation.

## Discussion

In the liver, ECM (Extracellular Matrix) makes up less than 3% of the relative tissue section area^22^. Hepatocyte survival and differentiation are heavily affected by cell–matrix interactions^23^. ECM is essential in regulating hepatocyte differentiation state and regenerative ability^24,25^. Past studies have documented hepatocyte dedifferentiation due to the loss of cell–matrix interactions^6,26^. Although the mechanism has not been fully elucidated, evidence suggests that cell attachment plays a crucial role in maintaining the *in-vitro* PHH culture^26^.

Methods to optimize PHH attachment have long been studied by primarily emphasizing matrix type or medium composition^27,28^. Freshly isolated PHH attached to various substrates such as polystyrene, collagen I, and Matrigel. Unfortunately, cryopreserved PHH showed a partial reduction in its attachment, preventing the cells from forming a confluent monolayer on most matrices, leading to cell death and a shortened cell culture period^4,29^. The low attachment hinders the pharmacological studies that require PHH to be cultured for an extended period. Fibronectin addition was proposed to be the solution to improve PHH attachment^30^. However, studies revealed that although fibronectin improved PHH attachment, the cells showed signs of over-spreading and over-time increase in stress fiber production^31^. Other studies also reported that adding apoptosis inhibitor Z-VAD-FMK improved PHH survival and attachment, although the mechanism that governs the PHH attachment process remains unknown^4^.

In this study, we found that BSA removal from the medium improved PHH attachment. The mechanism is unknown, but several studies hypothesized that BSA inhibits interactions between adhesion molecules and collagen^30,32^. Despite the benefit, BSA removal may negatively affect cell viability in the long run. Although BSA− treatment did not significantly change the cell parameters compared to the BSA-added medium, a wider variance was observed in multiple parameters, such as Albumin secretion and cell proliferation. BSA is commonly used in mammalian cell culture. BSA acts as an antioxidant and carrier for essential molecules in medium^33^. Its removal might be detrimental due to the absence of the stabilizing effect. Therefore, in long-term culture, we suggest that BSA− condition is only used for increasing early cell attachment (initial 4 h), continued with BSA+ medium for the rest culture.

Here, we reported that EPAC2 activation enhances cryopreserved PHH cell attachment and induces bile canaliculi formation in iPS-derived hepatocytes. cAMP binds to exchange protein directly activated by cAMP (EPAC) and directly activates Ras protein, leading to the regulation of cellular functions such as cell adhesion, cell–cell junction, and many more^9,12^. Furthermore, a putative isoform of EPAC, EPAC2C, is expressed specifically in the liver hepatocytes^14,34^. There is limited research focusing on this isoform. Hence further study needs to be conducted to elucidate its role in hepatocyte attachment and the maintenance of CYP3A4 activity in PHH.

Activation of cAMP signaling through IBMX and Forskolin treatment enhances the early attachment of PHH to the collagen matrix. In prolonged culture, the presence of IBMX and Forskolin in the commercially available medium for PHH maintained its maturity and morphology. IBMX works by increasing intracellular cAMP concentration by inhibiting the cAMP degradation enzyme (phosphodiesterase; PDE)^35^. Forskolin works synergistically with IBMX, activating adenylyl cyclase (AC), the enzyme responsible for cAMP synthesis^36,37^. Protein PKA is directly activated by cAMP, releasing active C subunits that phosphorylate metabolic enzymes and regulate various cellular processes, including cell adhesion^38,39^. The results suggest that cAMP signal transduction plays a role in initiating early PHH attachment and maintaining its function.

## Materials and Methods

### Human iPS cell lines

Our differentiation protocol used the RPChiPS771 iPS cell line (ReproCell)(Yoshioka et al., 2013). Undifferentiated iPS cells were maintained in AK02N StemFit media (Ajinomoto) on a cell culture dish (CellBIND; Corning) pre-coated with Synthemax II (Corning). For methionine deprivation, RPChiPS771 cells were cultured in a custom-made StemFit KA01 medium (a methionine-deprived medium; Ajinomoto).

### Media used for hepatic differentiation

**M1** consists of DMEM 4500 mg/L glucose (11995-073, ThermoFisher), supplemented with non-essential amino acids (NEAA; 11140050, ThermoFisher), L- glutamine (Gln; 16948-04, Nacalai Tesque), penicillin–streptomycin (PS; 49432-97, Kanto Chemical), 0.1 mM β-mercaptoethanol (β-ME; M6250, Sigma-Aldrich), B-27 Supplement (50 ×) (17504044, ThermoFisher), 100 ng/mL recombinant human activin A (GFH6, Cell Guidance Systems).

**M2** consists of Knockout DMEM/F12 (12660-012, ThermoFisher), supplemented with NEAA, L-Gln, PS, 0.1 mM β-ME, B-27 Supplement (50×), 10 ng/mL animal-Free BMP4 human recombinant (HZ-1045, Proteintech Group) and 10 ng/mL animal-Free human FGF-10 (AF-100-26, PeproTech).

**M3** consists of HBMTM (CC-3199, Lonza), supplemented with L-Gln, HCMTM SingleQuots (without GA1000) (CC-4182, Lonza), PS, 10 ng/mL recombinant human HGF (100-39, PeproTech) and 20 ng/mL recombinant human Oncostatin M (300-10, PeproTech).

**M4** consists of William’s medium E (49432-15, Kanto Chemical), supplemented with PS, L-Gln, HEPES (GB10, DOJINDO), insulin Human recombinant (49433-61, Kanto Chemical), transferrin human holo (49433-52, Kanto Chemical), 0.1% (w/v) bovine serum albumin (BSA; PRL68700, Proliant Biological), 10 ng/mL recombinant human HGF, 10 ng/mL recombinant human Oncostatin M, 1.5 μM 6-Bromoindirubin-3’-oxime (BIO; 6676296, BioGems), 10 μM Dexamethasone (Dex; 5000222, BioGems), 1 μM Calcitriol (3220632, BioGems), 10 μM Forskolin (6652995, BioGems) and 0.5 mM 3-Isobutyl-1-methylxanthine (IBMX; 2885842, BioGems).

### Chemicals

S-220 (Sp-Bnt-8-cAMPS) (B 046-05, Biolog), 007 (8-pCPT-2’-O-Me-cAMP) (17143, Cayman Chemical), 007-AM (8-pCPT-2’-O-Me-cAMP-AM) (SML-3072, Merck Millipore), ESI-09 (500506, Sigma Aldrich), 8-Br-cAMP (1140, Tocris Bioscience).

### Primary hepatocytes (PHHs)

Commercially available suspension type of PHH was purchased from Sekisui Xenotech (H1500.H15B, Female single donor suspension-type, Lot: H789, H877). The cells were kept at -150 °C until use.

### Differentiation of iPS cell-derived hepatocyte-like cells (iPS-hep)

RPChiPS771 cells were differentiated into the definitive endoderm (hiPS-endo). The resultant hiPS-endo were cryopreserved, as described (Nakai et al.^40^). For hepatocyte differentiation, cryopreserved day 3 hiPS-endo cells were freeze-thawed and plated onto rehydrated collagen vitrigel (CV) membrane 24 transwell inserts (ad-MED Vitrigel® 2, 08364-96, Kanto Chemical Co., Inc., culture area: 0.33 cm^2^/insert), at a concentration of 1 × 10^5^ cells/well in M2 medium supplemented with insulin-transferrin-selenium (ITS; 41400045, ThermoFisher) and Y27632 (251-00514, Wako). The medium volumes were 200 μL for the upper layer and 500 μL for the lower layer of the transwell. The media used for differentiation were: M2 for day 3-day 7, then changed to M3 for day 7- day 15, and then switched to M4 for day 15–day 31. Media in both upper and lower layers were replaced with fresh medium and growth factors every other day^40^.

### Matrigel Overlay on day 20 iPS-Hep

Matrigel overlay treatment was performed with 0.25 mg/mL Matrigel (354253, Corning) in culture medium overnight, followed by medium change on the next day.

### Real-time PCR analysis

RNA was extracted from iPS cells using the Cica geneus® RNA Prep Kit for Tissue (08057-96, Kanto Chemical). 2.5 µg RNA was reverse-transcribed using PrimeScript™ RT Master Mix (Takara Bio). The mRNA expression was quantified for real-time PCR analysis using SyberGreen on a StepOne Plus (Applied Biosystems, Foster City). The PCR conditions were as follows: initial denaturation at 95℃ for 30 s (s), then denaturation at 95℃ for 5 s, annealing, and extension at 60℃ for 30 s, for up to 40 cycles. Β-ACTIN and glyceraldehyde-3-phosphate dehydrogenase (GAPDH) were used as internal controls. Target mRNA levels were expressed as fold-changes against human adult hepatocytes (= 1). Primer details are listed in Supplementary Table S1.

### Measurement for CYP3A metabolites by LC–MS/MS

CYP3A activity in iPS-hep on day 30 of differentiation was evaluated. PHH plated for 4 h or 48 h were used as references. iPS-hep or PHH cultures were washed with warm William’s E media supplemented with PS, L-Gln, and Primary Hepatocyte Maintenance Supplements (CM4000, ThermoFisher). Assays were started by adding media containing 5 μM midazolam (Wako). The volume of media added was 500 μL to the PHH, 300 μL to the upper, and 600 μL to the lower chamber. 0.5, 1, and 2 h, 60 μL of supernatants were collected from the upper and lower chamber and kept at −80℃ until LC–MS/MS analysis of the metabolite, 1’-OH midazolam. For iPS-hep, midazolam was added to both the upper and lower chambers. Supernatants from both the upper and lower chambers were collected and combined. The collected supernatants were extracted by adding acetonitrile containing an internal standard, 1 ‘-OH midazolam ^13^C3. LC–MS/MS quantified the metabolites with the liquid chromatography Nexera I LC-2040C 3D (Shimadzu) and the mass spectrometer LCMS-8050 (Shimadzu), using Inertsil ODS-3, 2.1 × 33 mm, 3 µm column (GL Sciences) (Supplemental Table S2). The protein amount per well was quantified using the Bradford protein assay. Metabolite concentrations were normalized with protein amounts.

### PHH culture

Commercially available cryopreserved suspension-type Primary Human Hepatocytes (PHHs) (Sekisui Xenotech Lot no. H789 and H877, AMY > 4.0 × 10^6^ cells/vial) were thawed with ThawSTAR™ (Biocision), then decanted into OptiThaw medium (Sekisui Xenotech) then quantified in cell counting chamber (Invitrogen™ Countess II Automated Cell Countess; ThermoFisher). The cells were centrifuged at 100×*g* at 25 °C for 5 min. The cell pellets were resuspended with the treatment media, plated into 96-well plates at 75 µL / well (Collagen Type I-Coated, Flat-Bottom Microplates plate, Gibco™), and incubated at 37 °C for 4 h, followed by complete volume medium change. The cells were incubated for the required period with medium change every 24 h. Treatment media used were: Optiplate (K8200, Sekisui Xenotech), HCM (Hepatocyte Culture Medium SingleQuotsTM Kit) (CC-4182, Lonza), MM (Cellartis® Hepatocyte Maintenance Medium) (Takara Bio), LTMM (Cellartis Enhanced hiPS-HEP Long-Term Maintenance Medium) (Y30052, Takara Bio), Hep (Cellartis® Power™ Primary HEP Medium) (Y20020, Takarabio).

### CYP3A4 enzyme induction

For enzyme induction, PHH was plated with M4 media without BSA, BIO, IBMX and calcitrol, and supplemented with S-220. On day 2, media was replaced with William’s E medium without Phenol Red (A1217601, Gibco) containing Primary Hepatocyte Maintenance Supplements (CM4000 Life Technologies, Gibco) with 10 μM rifampicin, then incubated for 48 h. CYP3A activity assay was done, and RNA was extracted.

### Albumin and alpha-fetoprotein ELISA

Albumin secretion into the medium was quantified with Human Albumin ELISA Kit (E88-129, Bethyl). The process was performed according to the manufacturer’s guide. Alpha-fetoprotein ELISA was performed with Human AFP Sandwich ELISA Kit (KE00132, Proteintech) according to the manufacturer’s guide.

### Immunocytochemistry

Cells were fixed with 4% paraformaldehyde (Nacalai Tesque) in phosphate-buffered saline (PBS), permeabilized with 0.1% Triton X-100 (Nacalai Tesque), and blocked with 20% Blocking One (Nacalai Tesque) in PBST (0.1% Tween-20 in PBS). Antibodies were diluted in 20% Blocking One in PBST (0.1% Tween-20 in PBS). Cells were counterstained with 6-diamidino-2-phenylindole (DAPI; Roche Diagnostics). The following antibodies were used: goat anti-Albumin (ALB; A80-129A, 1:200, Bethyl Laboratories), Rabbit anti-Alpha-fetoprotein (Dako, Glostrup, Denmark), Rabbit anti-MRP2 (JA32-01, Invitrogen), Mouse anti-phospho-Histone H3 (Ser10) Antibody, clone 3H10 (05-806, Millipore), Donkey Anti-Goat IgG (H + L) whole antibody CF 568 Dye (20106, Biotium), Donkey Anti-Goat IgG (H + L) CF657 Dye (20048, Biotium), Donkey Anti-Rabbit IgG (H + L) CF488A (20015, Biotium), and Phalloidin-iFluor™ 488 Conjugate anti-AAT (23115, Bioquest).

### Live/dead assay

Live/Dead assay was performed with LIVE/DEAD™ Cell Imaging Kit (488/570) (R37601, Invitrogen) according to the manufacturer’s guide. Treated cells were visualized with MolecularDevice. Quantification was performed with MetaXpress.

### CDFDA assay

Cells were treated with 10 µM/ml CDFDA (Carboxy-DCFDA (5-(and-6)-Carboxy-2’,7’-Dichlorofluorescein Diacetate), C369, Invitrogen) medium for 10 min. HBSS washes (3x) were performed, followed by incubation with the culture medium for 15 min. Another round of medium change was performed. Cells were visualized with fluorescence confocal microscopy. The resulting image data were adjusted and quantified with a modified pipeline in CellProfiler to count the formed bile canalicular structure observed.

### Image acquisition, quantification, and editing

Image acquisition was performed with MetaXpress (Molecular Devices). The images and plate metadata were extracted with MetaXpress and analyzed using CellProfiler^41^ for cell identification and characterization using a modified pipeline. The database was then analyzed using the CellProfiler Analyst cell classifier module^42^. The data were processed with Microsoft Excel and GraphPad Prism. Image overlays were created with ImageJ and Adobe Photoshop.

### Statistical analysis

Individual data are shown or expressed as the mean ± standard deviation (SD). Cell attachment experiments were conducted with biological replicates (≥ 3) and repeated multiple times. The positive cell number in each image was obtained from CellProfiler Analyst results. The average positive cell number was calculated from 3 to 9 views (images) per well (treatment) × 3–5 wells (treatments) or the average of total positive cells in at least 3 wells. N = the total numbers of images or wells used for calculation, and are noted in the legend. The relative number of positive cells was obtained by dividing the average positive cell number (per image or well) by that in the control treatment.

Statistical analysis was performed using GraphPad Prism 7.0. Differences between groups are shown as *p < 0.05, **p < 0.01, ***p < 0.001 or ****p < 0.0001; analyzed by one-way ANOVA and Dunnett’s multiple comparisons test or two-way ANOVA, Sidak’s multiple comparisons tests.

## Supplementary Information

Supplementary Information.

## Data Availability

This paper does not report original database or any original code. Any additional information required to reanalyze the data reported in this paper is available from the corresponding authors upon request.
