# Supplementary material for: Activation of cAMP (EPAC2) signaling pathway promotes hepatocyte attachment
Source: Sci Rep. 2023 Jul 31;13:12352. doi: 10.1038/s41598-023-39712-3 (PMC10390557; doi:10.1038/s41598-023-39712-3)
Supplement: Supplementary file 1 — Supplementary Information. [file 41598_2023_39712_MOESM1_ESM.pdf]

Supplementary Information

Title:

Activation of cAMP (EPAC2) signaling pathway promotes hepatocyte attachment

List of authors:

Grace Aprilia Helena[1], Teruhiko Watanabe[2], Yusuke Kato[1], Nobuaki Shiraki[1,*], Shoen Kume[1,*]

Affiliations:

[1]School of Life Science and Technology, Tokyo Institute of Technology, 4259-B-25 Nagatsuta-cho, Midori-ku, Yokohama, Kanagawa 226-8501, Japan;.

[2]Life Science Laboratory, Technology and Development Division, Kanto Chemical Co., Inc., 21 Suzukawa, Isehara, Kanagawa 259-1146, Japan

[§]Corresponding authors:

Shoen Kume, Ph.D. skume@bio.titech.ac.jp

Nobuaki Shirak, Ph.D. shiraki@bio.titech.ac.jp

Table S1. Primer sequences used in Figure 1 and 5

Gene Symbol	Gene Name	Forward Primer Sequence	Reverse Primer Sequence
ABCB1	ATP-binding cassette family B member 1	GGAGCCTACTTGGTGGCACATAA	TGGCATAGTCAGGAGCAAATGAAC
ABCG2	ATP-binding cassette family G member 2	GGAGGCCTTGGGATACTTTGA	TCTATGAGTGGCTTATCCTGCTTG
AFP	alpha-fetoprotein.	AGAAACCCACTGGAGATGAACAGTC	GGCTGCAGCAGTCTGAATGTC
ALB	albumin	GCAGTGTCCATTTGAAGATCATGTA	TGCAACTGTGCATAATTTGTCTCC
APOA1	apolipoprotein A1	ACTGTGTACGTGGATGTGCTCAAAG	CACGCTGTCCCAGTTGTCAAG
APOB	apolipoprotein B	CAGTGAGCCAGCCTTGCAGTAG	GCTTTGGTGCAGGTCCAGTTC
CPS1	carbamoyl-phosphate synthase 1	AAGCCACATCAGACTGGCTCA	TCACTAGGTCAATGCTGCCATCTC
CYP1A1	cytochrome P450 1A1	AAACAGGGCCACATAGATGC	AGGGTCCTGGTTTGGCTAGT
CYP1A2	cytochrome P450 1A2	TGTTCAAGCACAGCAAGAAGG	TGCTCCAAAGATGTCATTGAC
CYP3A4	cytochrome P450 3A4	GAAACACAGATCCCCCTGAA	CTGGTGTTCTCAGGCACAGA
CYP7A1	cytochrome P450 7A1	GAGAAGGCAAACGGGTGAAC	GCACAACACCTTATGGTATGACA
DGAT1	diacylglycerol acyltransferase 1	AACACATGGAGGCCATAGCATAGAG	TCCTCGAAGATCACCTGCTTGTA
DGAT2	diacylglycerol acyltransferase 2	CCTGGCAAGAATGCAGTCAC	TTTGAAGACAACAAACGTGAACGAC
FABP1	fatty acid binding protein 1	AGTACCAACTGCAGAGCCAGGAA	ACTTTGGACCCAGCGGTGA
PPARA	peroxisome proliferator activated receptor-alpha	TCAGGCTATCATTACGGAGTCCAC	TTGAATGTCTTCAATGGGCTTCAC
UGT1A1	uridine diphosphate glucuronosyltransferase 1A1	TGGCTGTTCCCACTTACTGCAC	AGGGTCCGTCAGCATGACATC

Compound	Instrument	Column	LC condition				Q1 Pre Bias	Collision energy	Q3 Pre Bias	Monitoring ion (m/z)	
			Mobile phase		Gradient condition	Flow rate					
			A	B	% B / min	mL / min	V	eV	V	Precursor	Product
Midazolam	LCMS-8050 (SHIMADZU)	Inertsil ODS-3<br>2.1×33 mm, 3 μm<br>Cat: 5020-04411 (GL Science)	10 mM Ammonium acetate (pH 4.9)	Methanol	5-30-30-70-15-15 /0-0.21-1.70-3.20-4.99-5.00	0.40	-20.0	-53.0	-20.0	326.0855	291.0526
1'-hydroxy midazolam							-20.0	-53.0	-20.0	342.0804	168.0067
Hydroxy midazolam-[$^{13}C_3$]							-20.0	-53.0	-20.0	345.7400	171.0786

Supplemental Table S2. LC-MS/MS conditions for analysis

The compounds were identified and quanitified using LC-MS/MS with the condition indicated in the table.

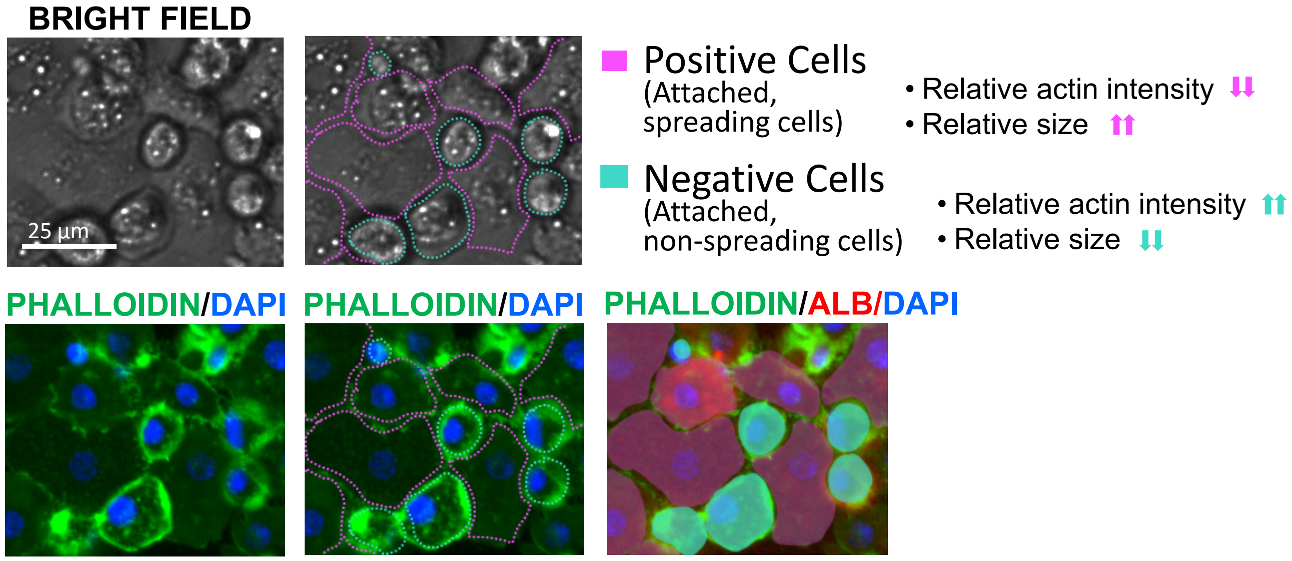

Supplementary Figure S1. Basis of manual classifications
The manual classification for positive/negative cells was based on a brightfield picture that showed "positive cells" (attached, spreading cells) exhibited lower relative actin intensity in comparison to "negative cells" (attached, non-spreading cells).

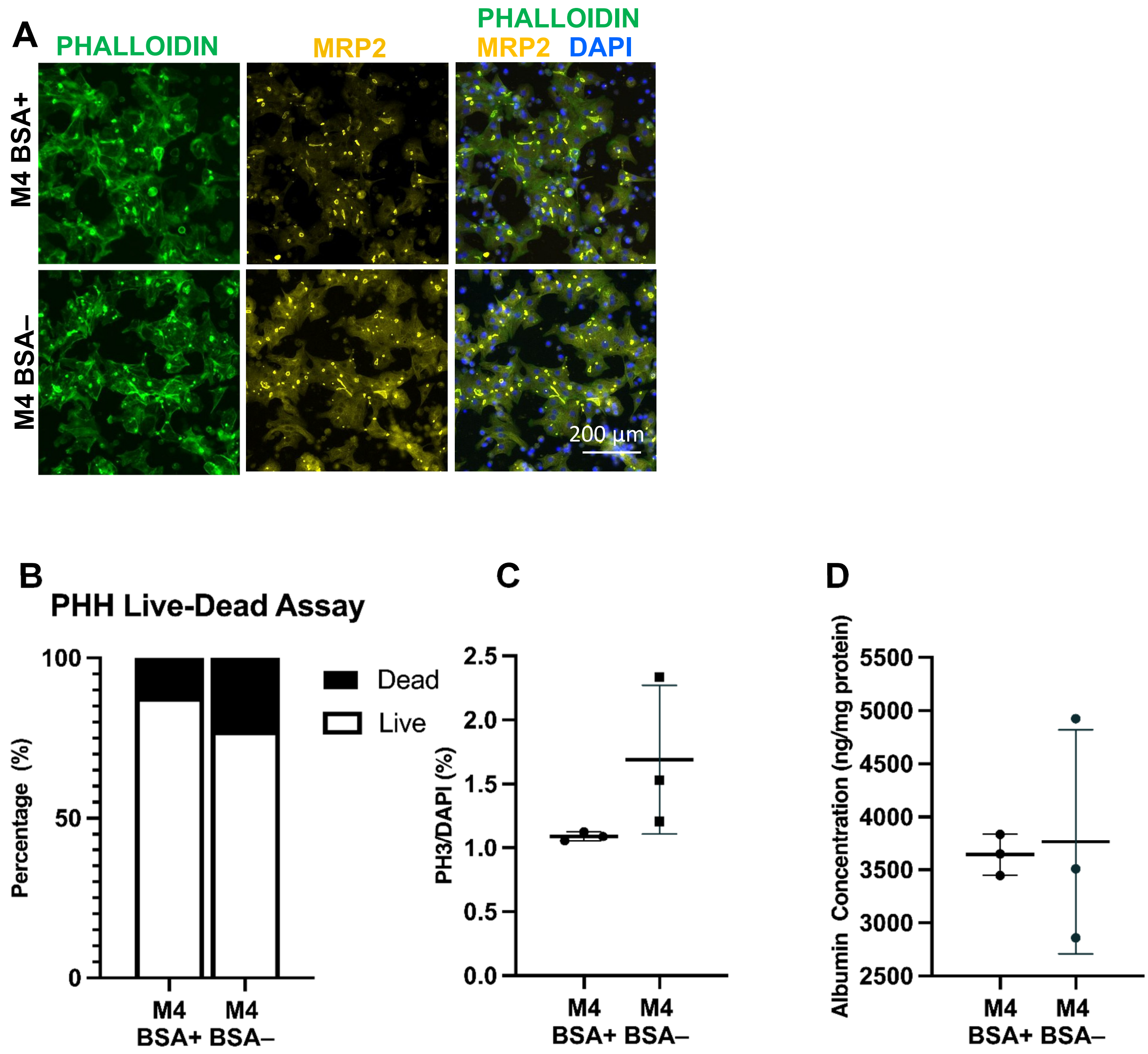

Supplementary Figure S2. Long-term cultured PHH in M4 BSA+/–

(A) Staining images of day 7 PHH cultured in M4 BSA+ and M4 BSA–, with Phalloidin (green), anti-MRP2 (yellow), and DAPI (blue).

(B) Live-dead assay of D7 PHH.

(C) PH3+ staining/DAPI for cell proliferation (n=3).

(D) Albumin secretion of D7 PHH (n=3) assayed by ELISA.

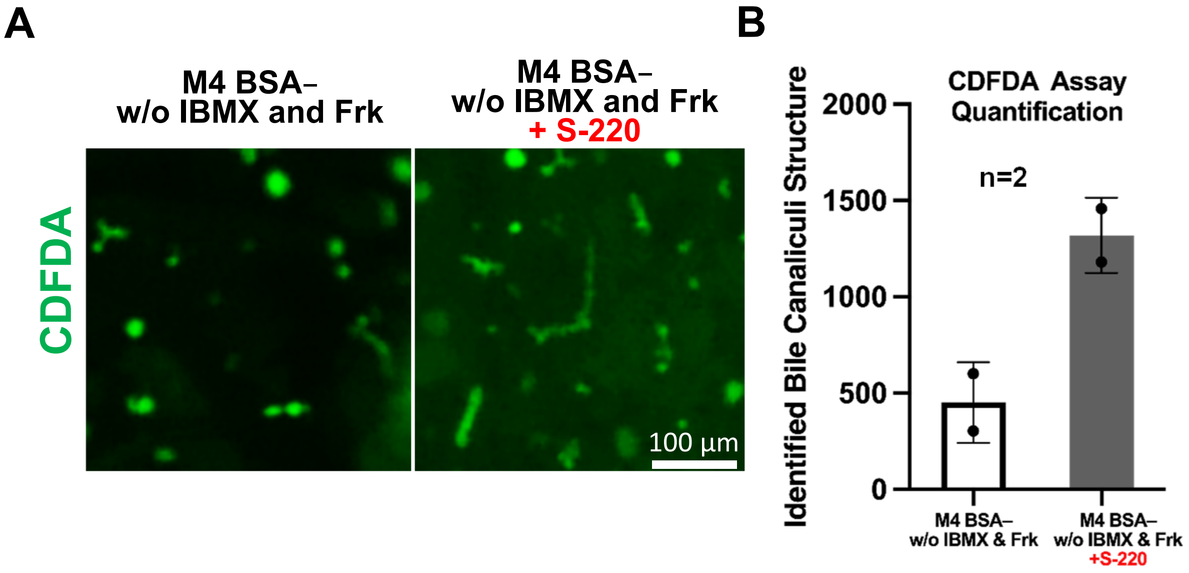

Supplementary Figure S3. EPAC2 activation in iPS-Hep improved bile canaliculi formation.

(A) CDFDA assay image of D31 iPS-Hep. Scale bar, 100 µm.

(B) Image quantification of the bile canaliculi structures (n=2).
